# Tension-Free Vaginal Tape versus Polyacrylamide Hydrogel Bulking Agent for Stress Urinary Incontinence: Patient Choice and Outcomes in Finland

**DOI:** 10.1007/s00192-025-06119-9

**Published:** 2025-03-29

**Authors:** Lotta Särkilahti, Camilla Isaksson, Tomi S. Mikkola

**Affiliations:** 1 Department of Obstetrics and Gynecology, Helsinki University Hospital, HUS, PO BOX 140, 00029 Helsinki, Finland; 2 Helsinki University, HUS, PO BOX 140, 00029 Helsinki, Finland

**Keywords:** Bulking agents, Finland, Hydrogels, Patient preference, Stress incontinence, Suburethral slings

## Abstract

**Introduction and Hypothesis:**

Since 2018 we have offered polyacrylamide hydrogel (PAHG) injection as an alternative to tension-free vaginal tape (TVT) for primary stress urinary incontinence (SUI). Our study aim was to investigate patient choice, demographics, re-treatments, and complications for these procedures.

**Methods:**

Patient demographics were collected from the Hospital Registry for women with primary SUI treated with TVT or PAHG, including patient age, body mass index, smoking status, obstetric history, and prior pelvic surgeries. Re-treatments and complications were collected at 2-year follow-up after each primary procedure.

**Results:**

Among 391 primary procedures, 55% (*n* = 217) of women chose the TVT and 45% (*n* = 174) the PAHG treatment, with similar patient demographics. Within 2 years, the re-treatment rates were 0.9% (*n* = 2) for TVT and 27.0% (*n* = 47) for PAHG (*p* < 0.001). Among patients undergoing re-treatment after primary PAHG, 57.4% (*n* = 27) opted for re-injection and 42.6% (*n* = 20) chose a mid-urethral sling. Three patients received TVT after two PAHG injection treatments. Complications occurred in 14.3% and 9.2% after TVT and PAHG respectively (*p* = 0.124). Complications after TVT ranged from Clavien–Dindo grades I–IIIb, with 4.1% of patients requiring reoperations, whereas PAHG complications were grades I–II with no reoperations. Including re-treatments, complication rates were 14.3% (TVT) and 10.9% (PAHG; *p* = 0.322).

**Conclusions:**

Similar clinical profiles in both TVT and PAHG groups suggest no specific demographic factors predict decision making. After a 2-year follow-up, the overall complication rates were similar, with PAHG associated with a higher likelihood of requiring re-treatment, whereas TVT carried a greater risk of severe complications. The re-treatment rates were lower than previously reported, indicating that actual patients are fairly satisfied with their primary choice.

## Introduction

Urinary incontinence severely affects quality of life [1]. The gold-standard invasive treatment for stress urinary incontinence (SUI) is a mid-urethral sling (MUS) procedure [2, 3]. Despite being a minor operation, MUS carries a risk of serious complications and long-term plastic mesh-related issues [4, 5]. Periurethral bulking with polyacrylamide hydrogel (PAHG) injection is a procedure without risk of serious complications, but it is less effective than MUS [6, 7]. Several studies have shown that effectiveness is just one of several factors influencing patients’ decisions when choosing treatments, highlighting the importance of shared decision making [8–10]. Thus, a woman should have an active role when choosing the treatment that best suits her personal expectations and values, together with the physician.

Most MUS surgeries in our university clinic are performed using the tension-free vaginal tape (TVT) technique, whereas the tension-free vaginal tape-obturator (TVT-O) technique is performed less frequently. We have offered PAHG as an alternative to MUS surgery for primary SUI treatment since 2018. We inform our patients based on the results from our randomized clinical trial (RCT) [7] that up to 90% are continent with TVT, whereas up to 70% are continent with PAHG after one or two injections. However, approximately one third of the PAHG-treated women will request a subsequent TVT procedure within 3 years post-treatment. Long-term complications associated with TVT, such as dysuria, pain, or tape erosion, are seen in 3–6% of cases [4, 5, 11]. Conversely, complications with PAHG are mostly transient, most commonly prolonged bladder-emptying time and urinary-tract infections [12].

We aimed to analyze women’s treatment choices between TVT and PAHG as the main outcome. Additionally, we assessed the patient demographics of the two groups, re-treatment rates, treatment complications, and identified possible patient factors that could predict treatment failure or success.

## Materials and Methods

This retrospective cohort study was conducted at Helsinki University Hospital. We analyzed patients who underwent a primary procedure for SUI with either TVT or PAHG bulking agent between 1 January 2019, and 31 December 2020. Patients defined as having primary SUI had not undergone any prior treatment, thus clarifying the distinction between untreated SUI and recurrent or secondary cases, where treatment had already been administered. The study was approved by the institutional review board of the Hospital District of Helsinki and Uusimaa (HUS/117/2022).

All patients were diagnosed with SUI based on a positive cough stress test and 2-day micturition diary to exclude patients with symptoms suggestive of urge urinary incontinence. All patients had conducted pelvic floor muscle training without sufficient relief and had requested invasive treatment. In clinical practice, our criteria for offering invasive treatment include having a body mass index (BMI) below 35, no more than second-degree urogenital prolapse, and no pregnancy plans, in accordance with the Finnish guidelines for invasive treatment of SUI.

All primary procedures were performed in an outpatient setting. TVT (TVT-Exact®; Gynecare, Ethicon, Johnson & Johnson, USA) was used as a mid-urethral sling, as previously described [2]. Briefly, the TVT was inserted under local anesthesia using approximately 100 ml of 0.25% lidocaine with adrenaline. Additional pain relief was given via intravenous fentanyl. Cystoscopy with 70º optic was performed during the operation to detect possible bladder perforation. The sling was adjusted to avoid retention using the cough test (200–300 ml saline in the bladder) allowing a few drops of saline to escape on vigorous coughing.

The PAHG injections (Bulkamid®; Axonics, USA) were performed as previously described [6]. Briefly, we used local anesthesia with periurethral lidocaine (10 ml) injections. Under endoscopic control at 1.5 cm from the vesicourethral junction, hydrogel was injected at the locations “10, 2, 5, and 7 o’clock” with the aim of the hydrogel cushions meeting at midline. Both patients with TVT and those with PAHG were discharged after a few hours of follow-up and successful micturition.

Before primary treatment, patients were informed of our 3-year RCT results [7], showing that treatment satisfaction (VAS ≥ 80) was reported in 95% of TVT cases and 68% of PAHG cases, although up to one-third of patients with PAHG may require re-injection. Complications were more frequently associated with TVT, with no complications in 57% of TVT cases and 76% of PAHG cases. Patients chose their treatment based on personal preferences. They were informed that they could contact our clinic within 6 months if they were dissatisfied with the treatment outcome or had procedure-related complications. Following 6 months, a referral from their general practitioner or private gynecologist was required for further evaluation.

We collected data from Helsinki University Hospital's quality registry (BCB Medical Ltd, disease specific platform for urogynecology v1.5.24, Finland) and medical records. The registry included information on treatments, re-treatments, complications, and pre-treatment Detrusor Instability Score [13] and Urinary Incontinence Severity Score (UISS) [14] results. Re-treatments and complications were tracked over a 2-year period following each primary treatment. Medical records provided details on patient age, BMI, smoking status, obstetric history, and previous pelvic surgeries.

Complications were documented when patients had experienced procedure-related symptoms requiring additional interventions or medical appointments, including discharge with an indwelling catheter, emergency-room returns, or a new doctor’s appointment owing to procedural symptoms.

These complications were categorized based on their manifestation and classified by severity using the Clavien–Dindo classification [15].

In the TVT group, one planned treatment was halted owing to bladder perforation, resulting in no tape insertion. This patient had no subsequent surgical procedures for SUI, but was included in our analysis under the intention-to-treat principle to maintain clinically relevant outcomes.

We analyzed data using IBM SPSS Statistics software (Version 29.0). Descriptive statistics were used to summarize patient demographics. Missing data were reported separately. Group comparisons were conducted using the independent samples *t* test for continuous variables and the Chi-squared test or Fisher's exact test, when appropriate, for categorical variables, with statistical significance set at a two-sided *p* value of < 0.05.

## Results

Our analysis included 391 primary procedures for SUI: 55% (*n* = 217) of the patients opted for the TVT procedure, and 45% (*n* = 174) chose PAHG injection. Patient demographics were similar between the two cohorts (Table 1). Both groups had a mean age of 53 years at the time of the primary procedure, and a mean BMI of 26 (Table 1).

**Table 1:** Patient demographics before primary treatment

	TVT, *n* = 217 (55%)		PAHG, *n* = 174 (45%)	
Age at surgery (years), mean ± SD	53 ± 10		53 ± 16	
Body mass index (kg/m^2^) mean ± SD	25.9 ± 3.7		26.1 ± 4.3	
Missing data *n*			2	
Smoking, *n* (%)	14 (6.5)		17 (10.0)	
Missing data, *n*	2		4	
Total vaginal deliveries, mean ± SD	2 ± 1		2 ± 1	
Patients with vaginal deliveries, *n* (%)	208 (95.9)		160 (92.5)	
Missing data, *n*			1	
Patients with previous pelvic surgery, *n* (%)	126 (58.1)		108 (62.1)	
Hysterectomy for prolapse	14 (6.5)		10 (5.8)	
Hysterectomy for nonprolapse indications	32 (14.7)		31 (18.1)	
Prolapse surgery (excluding hysterectomy)	38 (17.5)		33 (19.3)	
Other pelvic surgery	93 (42.9)		81 (46.6)	
Detrusor Instability Score, mean ± SD	5 ± 3		6 ± 3	
Missing data, *n*	12		23	
Urinary Incontinence Severity Score, mean ± SD	59 ± 17		59 ± 20	
Missing data, *n*	10		9	

*SD* standard deviation, *TVT* tension-free vaginal tape, *PAHG* polyacrylamide hydrogel

Four patients in the TVT group and 4 patients in the PAHG group had delivered by cesarean section only. Five patients in the TVT group and 9 patients in the PAHG group were nulliparous. Among the patients with a history of pelvic surgery, 49.2% in the TVT group and 53.7% in the PAHG group had undergone more than one pelvic surgery (Table 1).

Re-treatment and complication rates are presented in Table 2. Three patients died of unrelated causes during the 2 years following the primary procedure. The median time from the primary procedure to the first re-treatment was 10 months (range 5–15 months) after the TVT procedure and 7 months (range 1–19 months) after the PAHG injection.

**Table 2:** Re-treatment choices and complications within 2 years

	TVT, *n* = 217^a^ (55 %)		PAHG, *n* = 174 (45 %)		*p* value
Patients without re-treatment or complication visits, *n* (%)	185 (85.3)		114 (65.5)		<0.001
Re-treatment choice, *n* (%)	2 (0.9)		47 (27.0)		<0.001
Re-treatment with PAHG	2 (0.9)		24 (13.8)		<0.001
Re-treatment with TVT			18 (10.3)		<0.001
Re-treatment with TVT-O			2 (1.1)		0.197
Re-treatment with PAHG and subsequently TVT			3 (1.7)		0.087
Clavien–Dindo distribution of primary treatment complications, *n* (%)					
Grade I	17 (7.8)		8 (4.6)		
Grade II	5 (2.3)		7 (4.0)		
Grade IIIa	4 (1.8)				
Grade IIIb	5 (2.3)				
Primary treatment complications, *n* (%)	31 (14.3)		16 (9.2)		0.124
Bladder perforation	1 (0.5)				1.000
Acute urinary retention	12 (5.5)		9 (5.2)		0.876
Postoperative pain requiring pain medication	8 (3.7)		1 (0.6)		0.047
ER visit due to nausea/vomiting	1 (0.5)				1.000
Infection/hematoma requiring antibiotics	2 (0.9)				0.505
Urinary-tract infections	4 (1.8)		6 (3.4)		0.351
Dyspareunia	1 (0.5)				1.000
Vaginal tape exposure	3 (1.4)				0.257
Reoperation due to complications, *n* (%)	9 (4.1)				0.005
Patients with complications, including re-treatments, *n* (%)	31 (14.3)		19 (10.9)		0.322

*PAHG* polyacrylamide hydrogel, *TVT* tension-free vaginal tape, *TVT-O* tension-free vaginal tape-obturator

^a^One treatment was halted owing to bladder perforation, with no TVT inserted

Following primary TVT, only 2 (0.9%) patients requested re-treatment within 2 years, both receiving PAHG injection as re-treatment. One re-treatment was performed after the TVT tape was cut because of a complication.

After primary PAHG injection, 27.0% of women (*n* = 47) requested re-treatment. Among these patients, 57.4% (*n* = 27) chose re-injection, and 42.6% (*n* = 20) changed to MUS (TVT or TVT-O) as a second treatment. Of the patients with primary PAHG who were re-treated, 3 had experienced complications after their primary PAHG injection: 1 had a urinary-tract infection within 30 days post-surgery, and 2 had acute urinary retention requiring catheterization. Two underwent MUS, and 1 underwent re-injection as re-treatment. Three patients changed to TVT after two PAHG injections.

Similar patient demographics were observed among patients with primary PAHG who requested re-treatment (*n* = 47) compared with those who received no subsequent SUI treatments (*n* = 127). Additionally, the mean volume of the bulking agent used in the primary PAHG injection was similar between patients with PAHG requiring re-treatment (1.65 ml) and those who did not (1.58 ml).

Patients who underwent re-treatment after primary PAHG injection were compared in two groups: those who chose re-injection (*n* = 27) and those who opted for an MUS (*n* = 20). The re-injection group had a higher mean UISS of 67 before the primary procedure, compared with 53 in the MUS re-treatment group (*p* = 0.050). The mean bulking agent volume used in the primary PAHG injection was 1.56 ml in the re-injection group and 1.78 ml in the MUS re-treatment group (*p* = 0.100). The mean volume of bulking agent used in re-injections was 1.19 ml.

The primary treatment complication rates were 14.3% for patients in the TVT group, compared with 9.2% in the PAHG group (*p* = 0.124). When including re-treatment complications, the complication rates for treated patients were 14.3% and 10.9% respectively (*p* = 0.322), with neither difference reaching statistical significance (Table 2).

After primary TVT procedures, patients experienced complications ranging from Clavien–Dindo classification grades I–IIIb, showing a wider range of severity. Primary PAHG injections resulted in grades I–II complications, with none requiring reoperation.

A wide range of complications were detected after primary TVT procedures (Table 2). Reoperations due to complications (4.1%) were exclusive to patients who underwent the TVT procedure. The reasons for reoperations included acute urinary retention (*n* = 4), vaginal tape exposure (*n* = 3), pain (*n* = 1), and dyspareunia (*n* = 1). These reoperations involved the repair of vaginal tape exposure in 4 patients, tape loosening in 2 patients, tape cutting in 2 patients, and 1 patient had had both tape loosening and later tape cutting.

Comparing complications between TVT and PAHG treatments, a significant difference was observed in postoperative pain requiring additional pain medication, with an incidence of 3.7% in the TVT group compared with 0.6% in the PAHG group (*p* = 0.047; Table 2).

After re-treatments, 4 complications were documented. Of these, 3 complications occurred after re-treatment with TVT: 2 patients had urinary-tract infections, and 1 patient experienced pain. In total, 14.3% of patients treated with TVT as re-treatment after one or two PAHG injections (*n* = 21) experienced complications after TVT, a complication rate identical to those undergoing primary TVT treatment. Additionally, one complication occurred after PAHG re-injection, leading to an emergency room visit owing to nausea.

## Discussion

We show that approximately half of the women suffering from primary SUI will choose a less invasive procedure, although it is likely to be less effective. Interestingly, the patient demographics were similar in the two treatment groups. A previous study among women with primary SUI showed that decision making was not influenced by patient factors, such as age, BMI, smoking status, or previous laparotomies [8]. The severity of incontinence experienced by patients in our study, as assessed by the UISS, was not a factor when choosing the primary treatment option either. However, the retrospective analysis limits deeper understanding of patient choices, which reflect more than patient characteristics, with individual risk tolerance probably influencing the choice.

In the TVT group, we observed a re-treatment rate of 0.9%, consistent with other studies reporting retropubic MUS re-treatment rates of 0.7–1.2% after 1 year and 2.3–3.1% after 5 years [16, 17]. The PAHG group had a higher re-treatment rate of 27.0%, which aligns with a registry study comparing PAHG and MUS, showing PAHG re-treatment rates of 17.3% after 1 year and 27.6% after 5 years [16]. Among the patients with primary PAHG who requested re-treatment in our study, 57.4% chose an additional injection. Reported PAHG re-injection rates vary notably, ranging from 7 to 77% [18, 19]. However, it should be noted that there is likely a different indication for a PAHG re-injection if it is done soon (<6 months) after the primary treatment, aiming for a better primary outcome, whereas re-treatment after longer follow-up probably addresses the fading primary treatment effect.

In our previous non-inferiority RCT, involving a 3-year analysis of 110 patients randomized to TVT and 113 to PAHG, the primary endpoint was patient satisfaction and secondary endpoints included both objective and subjective outcomes. Of the women originally randomized to TVT, 3.3% received re-treatment, all with PAHG. Among patients randomized to PAHG, 32.3% had no re-treatments, 36.5% received one re-injection, 15.6% were re-treated with TVT, and 15.6% received TVT after two PAHG injections [7]. The more than 50% lower re-treatment rates that we observed indicate that patients experience an acceptable level of satisfaction with the outcomes of their chosen procedures in a real-life setting, although reasons for not contacting our clinic may vary. Thus, patient preferences and their realistic expectations are crucial when choosing the treatment. It appears that some women value a hierarchical approach to treatment for SUI, as shown in a previous study [8].

In our study, among the patients who requested re-treatment after PAHG, 43% changed to MUS (TVT or TVT-O) as their second treatment. A previous study also showed that 38% of patients who underwent primary PAHG preferred other subsequent SUI surgery over re-injection with PAHG [16]. Our analysis indicates that previous PAHG injections do not influence the risk for complications in subsequent TVT procedures. Supporting this, other studies have reported that prior urethral bulking injections do not impact the outcomes of subsequent MUS treatments [20–22].

We found that 14.3% of patients in the TVT group had complications, compared with 9.2% in the PAHG group. In our previous RCT, complications occurred in 44.6% of women in the TVT group and 19.6% in the PAHG group after 1 year, and 43.5% and 24.0% respectively after 3 years [6, 7]. The higher complication rates in the RCT may be attributed to the study design, which included proactive patient contact and a low drop-out rate, potentially revealing more complications than in other study settings. Including re-treatments in our results, the complication rates were 14.3% in the TVT group and 10.9% in the PAHG group, which should also be considered when assessing the overall risk of treatments.

In the current study, 4.1% of patients with primary TVT needed reoperation owing to postoperative complications, indicating a higher risk for severe complications compared with none in the PAHG group. Other studies have reported reoperation rates following retropubic sling insertion ranging from 0.9 to 1.6% after 1 year, and 1.2 to 4.8% in the long term [4, 5, 17, 23]. The primary reasons for reoperation after MUS were urinary retention and mesh exposure [4, 23], which were also observed in our study.

We detected that the most common complications following PAHG were acute urinary retention (5.2%) and urinary-tract infections (3.4%). Previous studies have reported the prevalence of acute urinary retention, ranging from 0 to 20% and urinary-tract infections between 1.6 and 40% [18, 19, 24]. The incidence of injection-site pain, which has been between 3.7 and 14% in previous studies [19, 24], was lower in our population (0.6%). Notably, we did not document de novo urgency, a complication reported in 0–10% of cases after PAHG injections [18].

The long-term risk of chronic complications associated with TVT [4, 11], along with the higher probability of needing more frequent re-treatment after PAHG [12], should also be discussed when choosing the treatment type, particularly for relatively young patients with SUI.

The strength of our study is that it includes a large population with a short- to medium-term follow-up time, yielding results based on data reflecting the real-life situation at a large urogynecology clinic. The procedures were also performed by experienced urogynecologists using the same bulking agent and technique determined in a previous RCT [7]. Furthermore, both procedures were performed in the same outpatient setting.

Our study also has limitations. First, our results are based on the information documented in the registries and dependent on patient reports to our clinic regarding dissatisfaction or complications. Furthermore, our data lack information on outcomes if patients visited a private clinic after the procedure, which may result in under-reporting of adverse events. However, the number of such patients is likely low as we provided patients with the possibility of contacting us directly.

Second, although patients with SUI may always choose the treatment as a part of shared decision making, the physician may have a role that could affect the decision, particularly in a re-treatment. As the study was conducted in Finland, where public health care covers treatment costs, the results may have limited applicability to settings in which financial factors impact choices.

Finally, we lacked data on urethral hypermobility, intrinsic sphincter function, and urodynamic testing before treatment, as these are not measured as part of our clinical practice for patients with uncomplicated primary SUI, in accordance with Finnish guidelines. However, as stated in the Cochrane review, in women with primary SUI invasive urodynamics do not provide further benefit in clinical outcomes over detailed clinical evaluation [25].

To conclude, our study indicates that patients choosing the TVT procedure versus PAHG treatment have similar demographic and clinical profiles. This highlights the fact that the decision-making process appears to be based on individual preferences and values. As patients have different goals and expectations when considering invasive treatment for SUI, patient-centered shared decision-making is increasingly important.

## Data Availability

The data generated and analysed in this study are not publicly available due to the new law of secondary use of social and health data in Finland.
